# Effectiveness of Comprehensive Intervention Programme on Quality of life, fatigue, self-efficacy, and psychosocial distress among head and neck cancer patients receiving radiotherapy

**DOI:** 10.1007/s00520-024-08381-x

**Published:** 2024-03-07

**Authors:** Shalini Ganesh Nayak, Anice George, Krishna Sharan, Baby S. Nayak, N. Ravishankar

**Affiliations:** 1 Medical Surgical Nursing, Manipal College of Nursing, Manipal Academy of Higher Education, Manipal, Karnataka India; 2 Radiotherapy & Oncology, KS Hegde Medical Academy, Nitte (Deemed to Be University), Mangalore, Karnataka India; 3 Department of Child Health Nursing Manipal College of Nursing Manipal Academy of Higher Education, Manipal, Karnataka India; 4 Department of Biostatistics, Vallabhbhai Patel Chest Institute, University of Delhi, New Delhi, India

**Keywords:** Head and neck cancer, Quality of life, Fatigue, Self-efficacy, Psychosocial distress, Radiotherapy, Well-being

## Abstract

**Purpose:**

The study aimed at evaluating the Effectiveness of Comprehensive Intervention Programme (CIP) on Quality of life (QOL), fatigue, self-efficacy, and psychosocial distress among Head and Neck Cancer (HNC) patients receiving radiotherapy treatment.

**Methods:**

Single-centre non-RCT time series study was conducted among 134 HNC patients (67 observed, followed by 67 subjected to CIP). FACT- H&N, FACT-F, Cancer Behavior Inventory and psychosocial distress scales were used to assess QOL, fatigue, self-efficacy, and psychosocial distress respectively. CIP was provided to the intervention arm twice a week during the course of radiation therapy along with the standard care; the control arm received only standard care. Data were collected before commencing radiotherapy, and post-test assessments were carried out at the end of radiotherapy treatment, and at 3 and 6 months after completion of radiotherapy.

**Results:**

Repeated measures ANOVA revealed a statistically significant improvement with CIP in QOL (*F* (1.917) = 454.103, *p* = 0.001), fatigue (*F* (2.106) = 183.775, *p* = 0.001), self-efficacy (*F* (2.429) = 190.861, *p* = 0.001), and psychosocial distress (*F* (2.288) = 290.105, *p* = 0.001) in the intervention arm.

**Conclusion:**

The CIP implemented to address multitude of issues in HNC patients receiving radiotherapy, proved to be effective in reducing the impact of treatment on QOL, fatigue, self-efficacy and psychosocial distress in HNC patients receiving radiotherapy.

## Introduction

Worldwide, Head and neck Cancers (HNC) are common at several regions. Globally, HNC accounts for more than 650,000 cases and 330,000 deaths annually [1]. These are group of cancers that develop in the tissues and organs located in the head and neck region. Radiotherapy (RT) has been used as primary source of treatment in all types of HNCs [2] and is often used in combination with surgery and/or chemotherapy [3]. RT is typically associated with significant side-effects, broadly classified as acute and chronic based on the time to manifestation. Though chronic toxicities are generally more concerning to the physicians, acute complications of RT such as mucositis, candidiasis, dysgeusia, and xerostomia significantly affect Quality of life (QOL) of HNC patients [4]. Treatment related side effects also lead to an inadequate nutritional intake, severe weight loss, lower disease related survival rates, patient related functional status and decreased QOL [5].

Despite advanced treatment methods in RT, the QOL is negatively impacted by HNC, even at early clinical stages [6]. QOL is important for HNC survivors and today as a study endpoint it is increasingly considered [7]. More than any other type of cancer, HNC patients place a high priority on their health-related Quality of Life (HR-QoL) [8]. The QOL of long-term HNC survivors is apparently poorer than that of the general population due to considerable functional limits caused by the illness and treatment [7]. Fatigue following RT among HNC patients is an unrecognised side effect [9, 10]. Fatigue stands out as one of the most prevalent side-effects of, and it impacts between 50 and 90% of individuals with HNC receiving RT [11, 12]. In HNC, fatigue may be directly related to malignancy, and to side-effects related to cancer treatment [10]. Burden of HNC is frequently manifested in psychosocial dysfunction, which may have a negative effect on QOL [13]. They have a wide range of psychological concerns throughout their illness, stressing the importance of therapeutic measures for preventing and treating psychosocial distress [14]. HNC patients also confront psychosocial distress such as anxiety and depression due to problems with salivation, eating and social contacts [13]. The high level of symptomatology such as mucositis, pain and xerostomia among HNC patients can manifest as social isolation, psychosocial distress and deteriorating QOL [15]. Patients are often anxious or fearful of suffering and death, uncertainty in the future and the treatment [16]. Throughout the illness trajectory, the HNC patients also have to cope with barrage of insults while facing debilitating treatment regimens, gruelling rehabilitation programmes and economic burdens. Hence, these psychosocial comorbidities in HNC patients must therefore be prevented, identified, and treated [14]. Depression is observed among 9.8 to 83.8% of patients, with a pooled estimated frequency of 63% (95% CI = 42–83) among HNC patients receiving radiation, with the heterogeneity (*I*^2^ = 97.66 percent; *p* < 0.001 which is statistically significant [17]. Enhancing the QOL of HNC patients by providing supportive care is the top priority in the healthcare [18]. The multifaceted nature of QOL issues among HNC patients also requires intervention with multiple components [19]. Considering such a wide spectrum of issues, more holistic interventions are needed to enhance the QOL, improve self-efficacy, and reduce fatigue and psychosocial distress of patients with HNC. Thus, this study aimed to evaluate the benefit of early implementation of Comprehensive Intervention Program (CIP) in improving QOL and self-efficacy, and reducing fatigue and psychosocial distress among HNC patients.

## Materials and methods

A single centre non RCT time series study using pre and post-test design was conducted among 134 (67 in each arm) HNC patients receiving RT, selected by purposive sampling technique. The sample size was estimated by considering the QOL as the primary outcome variable. The following formula of comparison of means was used to find the sample size.

$$n=\frac{2{({Z}_{1-\alpha /2}+{Z}_{1-\beta })}^{2}{\sigma }^{2}}{{d}^{2}}$$

*n* = needed minimum sample size for each arm, Z_1-α/2_ = 1.96; α = 0.05, Z_1-β_ = 0.84 with 80% power, σ = population standard deviation of the primary outcome variable (SD = range/4; 156/4 = 39) and *d* = 20; i.e., clinically significant difference.

The data were collected between October 2020 and August 2022 from a tertiary care hospital of South India after obtaining clearance from Institutional Ethical Committee (IEC: 114/2019) and registration under Clinical Trial Registry of India (CTRI/2019/04/018820). The data from control arm was collected first and then from intervention arm, to avoid contamination. HNC patients in stage III, stage IV_a_ and stage IV_b_ receiving RT with or without chemotherapy with the curative intent, age between 40 and 80 years and Eastern Cooperative Oncology Group (ECOG) performance [20] status grade ≤ 1 were included in the study. Patients with early-stage vocal cord malignancy, with distant metastasis, secondary HNC, previous history of cancers and cancer treatments, with history of mental health disorders and patients having active infections or other comorbidities that could directly or indirectly impact on their QOL, were excluded. Control arm received standard care and intervention arm received CIP along with standard care. Follow-up was done for both arms at the end of intervention, three and six months after completion of intervention.

The baseline data on sample and disease characteristics were recorded on a proforma. QOL and fatigue were assessed by Functional Assessment of Cancer Therapy: Head & Neck (FACT-H&N) [21] & Functional Assessment of Cancer Therapy: Fatigue (FACT-F) [22] respectively after obtaining required permission from FACIT.org. Self-efficacy was measured through Cancer Behavior Inventory (CBI)—Brief form [23] with approval from PsychooncologyNDU.Wordpress.COM. A psychosocial distress scale was developed by the researcher [24] after extensive literature review [17]. The tool has 21 items with three responses “never,” “sometimes,” and “always.” The maximum possible score was 42 and minimum score was zero. Higher the score, lesser the psychosocial distress and vice versa. The content validity index of the tool was 0.98 and the reliability computed through Cronbach’s alpha was 0.87. The data were collected before starting radiotherapy (before CIP), at the end of radiotherapy (end of CIP), three and six months after the completion of radiotherapy.

### Comprehensive intervention programme

We designed a psychosocial interventional package intended for improving the QOL among HNC patients receiving radiotherapy. The researcher consulted international and national experts in the subject for the development of appropriate intervention. Many of the unanswered questions from the need analysis and review of literature were discussed with professionals in the field before developing a culturally specific CIP, suitable for the research setting. It had five components: Seated exercise therapy, Reminiscence therapy, Board based games, educational sessions and Information booklet. Seated exercise therapy included a group of exercises performed to promote the wellbeing by sitting on an immovable chair with flat seat. This included warm-up exercises for five minutes such as feet taps, shoulder exercise, back twist, leg marches, and chair marches; moderate intensity exercises for 10 to 15 min, such as thigh stretches, calf stretches, upper back strengthening, arm strengthening, chest strengthening, hip strengthening, thigh strengthening; and finally, cool-down exercise for five minutes such as neck rotation, hand presses, stretching hands and legs, pelvic floor exercises, and walking around for one to two minutes. Reminiscence therapy was centred on telling stories depending on the session's focus. It was a “life review,” where the HNC patient looked back at his/her life and reflected the past pleasant experiences by vocally recalling to the questions related to the themes such as childhood, school days, family, occupation, marriage and children, visits and events, feelings about self, objects, festivals and holidays, entertainment, personal pictures and sharing the experience in the group. These topics were mentioned in cards; the participants were asked to select a card, and were then encouraged to share their memories with other patients, with particular emphasis on discussions that are pleasant to them; these were organized group sessions lasting 20 to 30 min. Board-based games included indoor, table top traditional games of India, such as carrom, snakes and ladders, monopoly, Ludo and Chinese checkers, which were played for 30–40 min. Seated exercise therapy, Reminiscence therapy and Board based games were administered twice a week for the duration of radiotherapy treatment. Educational session was prepared by considering the various aspects such as disease specific information of treatment modalities, detailed description of RT, preparation for RT, chemotherapy, common side effects of RT, modes of communication in difficult verbal communication, nutritional management, management of pain and other symptoms of RT and anticipatory guidance. This session was delivered to the HNC patient along with family member for the duration of 45 min prior to RT and during the fourth week of RT. The information booklet was tailored based on the needs of the HNC patients after RT, supplemented with appropriate pictures. The content of the booklet included oral care, personal hygiene and care of RT site, nutritional management, prevention of infection, distraction from ruminative thoughts and other discharge instructions. The intervention was validated by nine subject experts and pilot tested among five HNC patients for the feasibility. The intervention was implemented adhering to the protocol by a single researcher throughout to maintain the consistency. A checklist on duration and missing of sessions was maintained by the researcher to assess the adherence.

## Results

**Fig. 1:**
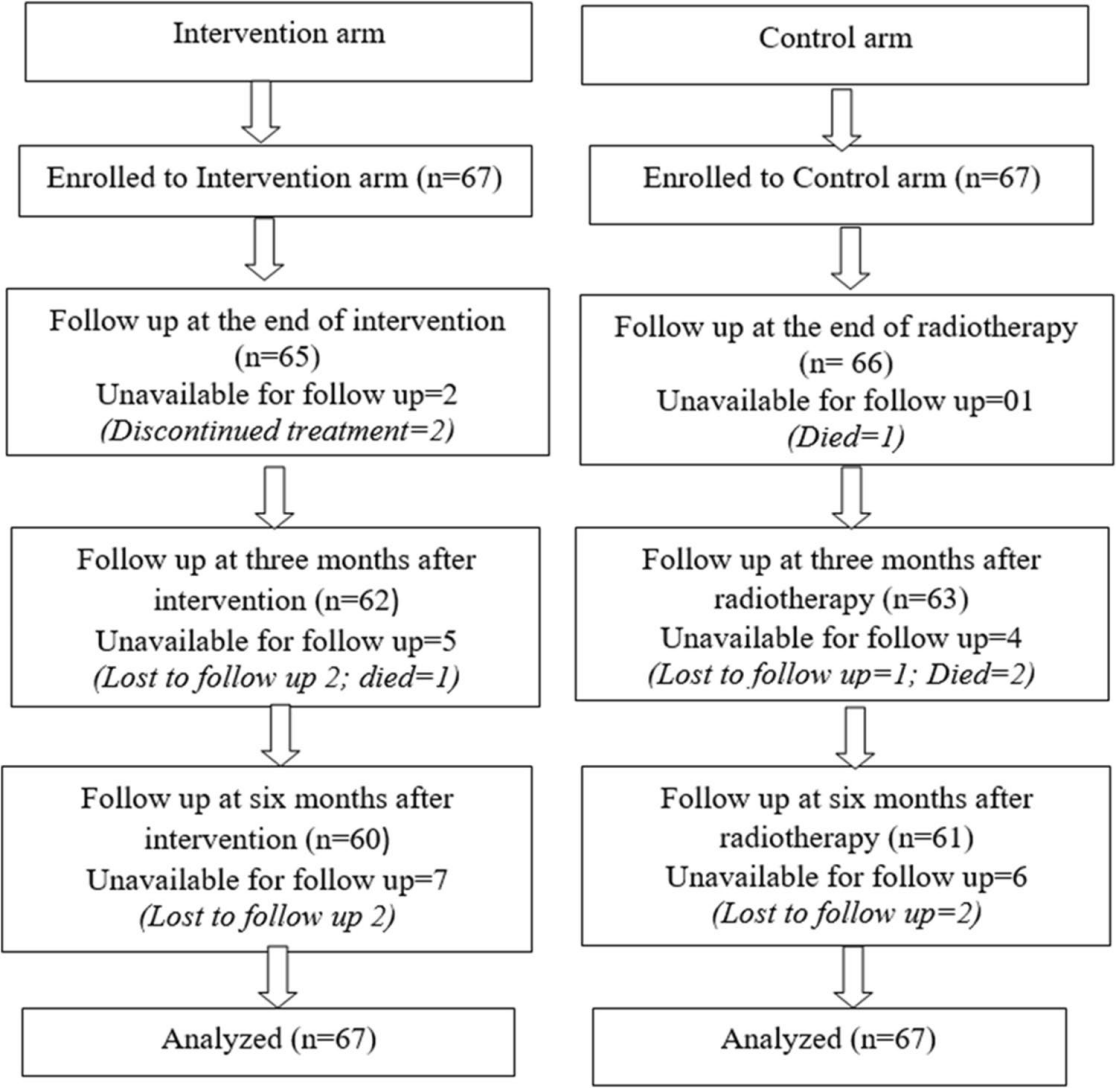
Flow diagram of study participants

The coded data were entered and analysed by Statistical Package for the Social Sciences (SPSS) version 16. Patients enrolled in the study (134) and the participants’ attrition is presented in Fig. 1. Repeated measures ANOVA with general linear model was computed and the last recorded value was substituted for the intention to treat (ITT) analysis. To find whether difference occurred is true Bonferroni correction and pairwise comparison was done. For the lost to follow-up data, LOCF (last observation carry forward) was used.

The demographic findings showed that most participants belonged to the age group of 51–70 years, i.e., 44 (65.6%) from intervention arm and 49 (73.1%) from control arm, with the mean ± SD age of 56.1 ± 9.4 years and 57.6 ± 8.3 years respectively. Sample had male preponderance, with 58 (86.6%) and 57 (85.1%) in intervention and control arm respectively. Approximately half of the sample, i.e., 31 (46.3%) from intervention arm and 35 (52.3%) from control arm had only primary education. Predominant site of cancer was oral cavity, i.e., 34 (50.7%) and 40 (59.7%) of the participants from intervention and control arm respectively. Regarding the stage of cancer, 37 (55.2%) of the participants from intervention arm and 39 (58.2%) from control arm were diagnosed to have the Stage IVa disease. Chi-square computed to assess the homogeneity of intervention and control arm on baseline demographic characteristics and disease related variables showed that the groups were similar (*p* > 0.05) (Table 1 and 2).

**Table 1:** Chi-square to assess the homogeneity of baseline socio-demographic variables

Socio-demographic Variables	Intervention Arm (*n* = 67)		Control arm (*n* = 67)		*P* value
	*f*	%	*f*	%	
Mean age (mean ± SD)	56.1 ± 9.4		57.6 ± 8.3		
Age group (in years)					
40–50	20	29.9	14	20.9	0.37
51–60	22	32.8	31	46.3	
61–70	22	32.8	18	26.8	
71–80	03	4.5	04	6.0	
Gender					
Male	58	86.6	57	85.1	0.80
Female	09	13.4	10	14.9	
Marital status					
Living with spouse	61	91.0	60	89.6	0.95
Widow/widower	05	7.5	06	8.9	
Divorced	00	00	00	00	
Unmarried	01	1.5	01	1.5	
Educational qualification					
Primary	31	46.3	35	52.3	0.75
High school	26	38.8	22	32.8	
Pre-University and above	10	14.9	10	14.9	
Occupation					
Agriculture	14	20.9	16	23.9	0.19
Business	03	4.5	10	14.9	
Cooli	16	23.9	14	20.9	
Others	19	28.4	19	28.4	
Nil	15	22.3	08	11.9	
Type of family					
Nuclear	41	61.2	37	55.2	0.33
Joint	15	22.4	12	17.9	
Extended	11	16.4	18	26.9	
Family income per month in rupees					
≤ 6000	23	34.3	21	31.3	
6001–18,000	34	50.7	27	40.3	0.29
18,001–30,000	06	9.0	07	10.4	
30,001–45,000	02	3.0	06	9.0	
45,001–60,000	02	3.0	03	4.5	
Above 60,000	00	00	03	4.5	
Type of diet					
Vegetarian	10	14.9	10	14.9	
Non-vegetarian	00	00	00	00	1.0
Mixed	57	85.1	57	85.1

**Table 2:** Chi-square to assess the homogeneity of baseline disease related variables

Disease related variables	Intervention Arm (*n* = 67)		Control arm (*n* = 67)		*P* value
	*f*	%	*f*	%	
Site of cancer					
Pharynx	21	31.3	15	22.4	0.17
Larynx	05	7.5	01	1.5	
Oral cavity (Buccal mucosa/ Tongue/ Alveolus)	34	50.7	40	59.7	
Others	07	10.4	11	16.4	
Stage of cancer					
Stage III	13	19.4	12	17.9	0.94
Stage IVa	37	55.2	39	58.2	
Stage IVb	17	25.4	16	23.9	
Duration of illness					
3 months and less	18	26.9	25	37.3	0.20
More than three months	49	73.1	42	62.7	
Treatment modality					
Only radiation therapy	05	7.4	04	6.0	0.34
Radiation therapy & Chemotherapy	32	47.8	38	56.7	
Surgery, Radiotherapy and chemotherapy	17	25.4	09	13.4	
Any other treatment (surgery + radiotherapy)	13	19.4	16	23.9	
Radiotherapy dose					
60 Gy/30#/6 weeks	19	28.4	22	32.8	0.46
66 Gy/33#/6.5 weeks & 70 Gy/35#/7 weeks	48	71.6	45	67.2	
Co-morbid illness					
Yes	10	14.9	16	23.9	0.19
No	57	85.1	51	76.1	

### Description of baseline QOL, fatigue, self-efficacy and psychosocial distress

Baseline mean and standard deviation of QOL, fatigue, self-efficacy and psychosocial distress are presented in Table 3. Independent sample ‘t’ test computed shows that the data were not significantly different in both the arms at baseline and the groups were comparable.

**Table 3:** Comparison of intervention and control arm participants on baseline QOL, fatigue, self-efficacy and psychosocial distress

								*N* = 134
Outcome variables	Intervention arm (*n* = 67)		Control arm (*n* = 67)		95% of CI		‘t’ value	*p* value
	Mean	+ SD	Mean	+ SD	LL	UL		
QOL	85.52	18.75	93.01	18.60	1.09	13.87	2.32	0.12
Fatigue	36.87	10.39	38.57	9.08	-1.63	5.03	1.00	0.32
Self-efficacy	70.31	13.88	72.98	12.76	-1.88	7.23	1.15	0.23
Psychosocial distress	25.79	6.34	28.10	6.09	0.19	4.43	2.15	0.33

### Description of pre and post-test QOL, fatigue, self-efficacy and psychosocial distress

Pre and post-test QOL, fatigue, self-efficacy, and psychosocial distress are shown in Table 4.

**Table 4:** Pre and post-test QOL, fatigue, self-efficacy, and psychosocial distress among the participants of intervention and control arm

								*N* = *134*	
Outcome variables	Study arms	Baseline		At the end of intervention		After three months		After 6 months	
		Mean	+ SD	Mean	+ SD	Mean	+ SD	Mean	+ SD
QOL domains									
Physical wellbeing (0–28)	Intervention arm	15.70	4.31	18.00	3.24	24.09	1.87	26.20	1.62
	Control arm	16.91	2.81	10.62	5.07	21.52	2.27	24.89	2.49
Social wellbeing (0–28)	Intervention arm	17.22	5.53	20.49	2.88	22.23	5.74	21.73	2.24
	Control arm	18.52	4.95	18.48	3.84	20.31	2.76	20.81	2.92
Emotional wellbeing (0–24)	Intervention arm	18.32	4.10	19.90	1.98	22.51	1.41	23.45	1.04
	Control arm	19.68	2.85	14.00	4.05	20.82	1.98	22.31	2.21
Functional wellbeing (0–28)	Intervention arm	14.92	6.04	14.90	3.56	23.74	3.10	25.28	2.97
	Control arm	16.85	6.72	7.45	4.09	18.30	3.86	22.44	3.19
H&N subscale (0–40)	Intervention arm	19.34	6.10	20.18	2.65	29.66	3.02	33.53	3.12
	Control arm	21.04	6.87	12.22	3.75	26.60	3.96	31.38	4.18
FACT-H&N total (QOL) (0–148)	Intervention arm	85.52	18.75	93.49	8.67	122.27	8.57	130.20	8.25
	Control arm	93.01	18.60	62.78	11.49	107.57	9.18	121.83	10.96
Fatigue	Intervention arm	36.86	10.39	34.87	5.22	44.59	4.50	47.07	3.78
	Control arm	38.56	9.08	20.45	7.99	38.39	6.49	43.59	5.69
Self-efficacy	Intervention arm	70.31	13.88	89.20	8.14	97.19	5.35	101.76	4.46
	Control arm	72.98	12.76	61.16	17.11	86.82	13.47	92.50	11.03
Psycho-social distress	Intervention arm	25.79	6.33	27.72	3.64	37.48	2.49	39.51	2.49
	Control arm	28.10	6.09	17.83	5.18	31.19	4.05	35.26	4.62

As shown in Table 4. the mean (± SD) of QOL score of intervention arm was 85.52 (± 18.75) and control arm was 93.01 (± 18.60), indicating that the baseline QOL was apparently better in control arm. Mean scores in all the domains of QOL had improved slightly in the participants of intervention arm at the end of intervention except for functional wellbeing. The mean (± SD) scores in all the domains steadily improved among the participants of both arms from the end of intervention to 3 and 6 months. The intervention arm maintained higher mean scores compared to control arm in all the domains of QOL at follow up observations.

Fatigue in both intervention and control arm worsened from baseline to the end of intervention. However, it was comparatively less impacted in the intervention arm. Mean (± SD) scores of self-efficacy scores improved among the participants of intervention arm from baseline to the end of intervention i.e. 70.31 (± 13.88) to 89.20 (± 8.14) in comparison to control arm. Decrease in the mean (± SD) scores of psychosocial distress in the control arm from baseline 28.10 (± 6.09) to 17.83 (± 5.18) was observed indicating more psychosocial distress among the participants at the end of radiotherapy comparing to intervention arm. There was steady increase in the mean (± SD) score of fatigue, self-efficacy and psychosocial distress scores at 3 and 6 months among the participants of both arms. The intervention arm maintained higher mean (± SD) scores throughout the follow up observations (Table 4).

### Effectiveness of comprehensive intervention programme

Findings revealed statistically significant difference in QOL (*F*_(1,119)_ = 77.507, *p* = 0.001, ${N}_{p}^{2}$= 0.394), fatigue (*F*_(1,119)_ = 45.067, *p* = 0.001, ${N}_{p}^{2}$= 0.275), self-efficacy (*F*_(1,119)_ = 73.762, *p* = 0.001, ${N}_{p}^{2}$= 0.385) and psychosocial distress (*F*_(1,119)_ = 101.788, *p* = 0.001, ${N}_{p}^{2}$= 0.461) between the subjects of intervention and control arm. The effect size of QOL (0.394), fatigue (0.275), self-efficacy (0.385) and psychosocial distress (0.461) between the arms shows moderate statistical significance. The interaction effect (time X group) of QOL (*F* (1) = 5.47, *p* = 0.021) and psychosocial distress (F (1) = 13.090, *p* = 0.001) was significant, indicating that the group changed over time and the change was different across the groups. However, it was not significant for fatigue (*F* (1) = 1.271, *p* = 0.262) and self-efficacy (*F* (1) = 2.671, *p* = 0.105). The interaction effect (time X group) was considered to determine the significant variation in the change across the groups (Table 5).

**Table 5:** Repeated Measures ANOVA on QOL, fatigue, self-efficacy, and psychosocial distress scores between intervention and control arm HNC patients

					*N* = *134*
Variables	Mean square	*F* value	df	*p* value	${N}_{p}^{2}$
QOL					
Between group (*n* = 134)	16417.257	77.507	1,119	0.001	0.394
Within group (*n* = 67)	94583.006	454.103	1.917	0.001	0.792
Time X group	1188.01	5.47	1	0.021	0.044
Fatigue					
Between group (*n* = 134)	3687.780	45.067	1,119	0.001	0.275
Within group (*n* = 67)	10088.235	183.775	2.106	0.001	0.607
Time X group	69.367	1.271	1	0.262	0.011
Self-efficacy					
Between group (*n* = 134)	15131.302	73.762	1,119	0.001	0.385
Within group (*n* = 67)	23916.695	190.861	2.429	0.001	0.618
Time X group	323.400	2.671	1	0.105	0.022
Psychosocial distress					
Between group (*n* = 134)	2479.177	101.788	1,119	0.001	0.461
Within group (*n* = 67)	7259.451	290.105	2.288	0.001	0.709
Time X group	331.153	13.090	1	0.001	0.099

*df* degree of freedom, ${N}_{p}^{2}$ Partial Eta Squared (effect size)

**Fig. 2:**
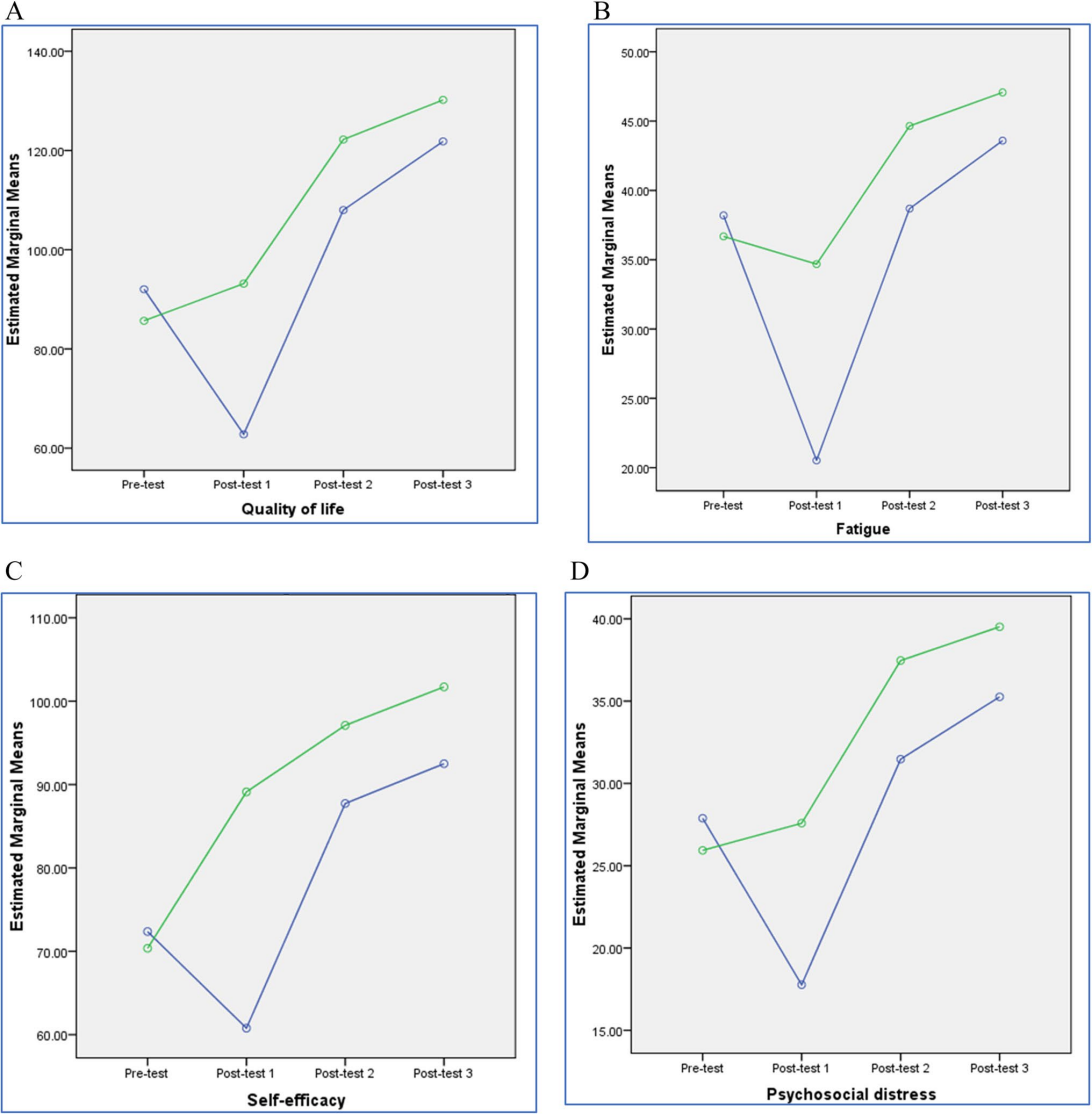
Profile plot showing mean QOL (**A**), fatigue (**B**), self-efficacy (**C**) and psychosocial distress (**D**) scores

The difference in QOL, fatigue, self-efficacy and psycho-social distress scores was plotted in Fig. 2.

## Discussion

In this study, effectiveness of CIP was evaluated and demonstrated to be effective in improving the QOL, decreasing fatigue, improving self-efficacy and reducing psychosocial distress of HNC patients. These findings are in line with the results of a study on effectiveness of a “Comprehensive oral care programme” by Lee et.al., among HNC patients undergoing RT, which showed statistically significant (P < 0.05) lower speech problems (*p* = 0.034) and swallowing problems (*p* = 0.035) in the domain of QOL [25]. Similar findings were identified in a study conducted by Lin et al., to assess the effectiveness of “mobile oral care app,” which had an educational content to improve QOL. The results of this study showed significant improvement in QOL among the participants of intervention group (*p* < 0.001) [26]. The literature supports the benefits of exercise therapy and psychosocial programmes in improving the QOL among HNC patients [27, 28]. A systematic review conducted by Lynch et al., to assess the “effectiveness of physical activity interventions” showed improvement in QOL along with other symptoms such as pain and sleep [29]. Findings of another systematic review summarised from 16 research articles by Capozzi et al., to assess the effectiveness of physical activity on QOL among HNC patients also demonstrated feasibility and safety of physical interventions along with improvement in QOL and physical activity [30]. Systematic review on effectiveness of “psychological interventions” on QOL by Calver et. al., demonstrated inconclusive results due to insufficient data (Calver, Tickle, Moghaddam, Biswas, & Macmillan, 2018). Enhancing the QOL among HNC patients requires a comprehensive and multidimensional approach. Hence, it is quiet challenging to conclude the best and most effective intervention and time of implementation owing to the heterogeneity in sites of cancer, use of diverse populace, variation in type and rigour of interventions used, non-comprehensiveness and differing outcomes.

In the current study, CIP demonstrated effective in reducing the fatigue. The findings of this study are supported by few of the published research studies. Grote et al., conducted a pilot randomised controlled trial at Germany among 20 HNC patients receiving RT with “progressive resistance training” as an intervention. They reported the intervention to be beneficial in reducing the general fatigue, though not statistically significant (*p* = 0.393) [31]. A systematic review by Lynch et al., and Capozzi et al., also reported reduction in fatigue among HNC patients [29, 30]. Exercise training during anticancer therapy has shown improvement in physical function and reduction in cancer related fatigue. Hence, exercise should be regarded as a complementary therapy to RT in order to reduce treatment-related side effects and speed up recovery [32]. Patients diagnosed with HNC have low level of physical activity and sedentary behaviour [33]. Low- to moderate-intensity exercise, such as sitting exercise, can help to reduce fatigue over time [34]. Among older adults, seated exercises can have positive effect on cognition, strength, activity and QOL [35].

As the HNC patients suffer from multitude of problems, the interventions should be focused on effective self-management to enhance self-efficacy. However, scientific evidence on interventions focussing on the improvement of self-efficacy is minimal. Functional restrictions attributed by HNC may lead into psychosocial distress. CIP was effective in improving self-efficacy and decreasing psychosocial distress, as it combined multiple components and addressed the diverse needs of HNC patients. Reminiscence therapy used in CIP proved to significantly reduce anxiety and depression, in addition to improving social functioning, mental and physical health [36]. It is an effective and safe nursing intervention in improving self-esteem, emotional and physical wellbeing [37] and decreasing adaptation difficulties among elderly [38]. Board based games have proven to be effective in improving physical activity, cognitive function, enhancing motivation, interpersonal interaction and are thus regarded as effective complementary therapy to improve clinical symptoms [39]. Educational interventions also improve the physical symptoms and psychological outcomes [40]. These interventions can also reduce the fatigue and related symptoms, decrease anxiety and improve QOL [41]. While our study was conducted in India, its implications extend beyond the borders of our nation. Although our data is rooted in the Indian context, the patterns and trends in HNC are align closely with reports from other countries in Aasia facing comparable demographic transitions and public health challenges [42]. This suggests a degree of generalizability of our results to countries with similar profile. However, we acknowledge the need for further research to confirm the generalizability of our findings.

## Implications

HNC patients require multimodal treatment modalities and oftentimes, structural, functional, and aesthetic losses result with HNC treatment. From the time of diagnosis, treatment to post treatment follow-up, it is a difficult and lengthy journey which makes HNC one of the most painful cancer experiences mentally and emotionally. The clinical focus during treatment and post-treatment is changing with the emphasis on identifying and minimising treatment side effects and rehabbing the functional deficiencies. Burden of physical symptoms impair the physical, emotional, social and functional wellbeing impacting the QOL. Supporting patients and providing psychosocial services constitute crucial elements of cancer care. Comprehensive cancer centres must offer a range of these services to ensure they deliver high-quality and comprehensive care to cancer patients. The delivery of these services initiates upon diagnosis, extends throughout cancer treatment, and transitions accordingly into survivorship or end-of-life stages [43]. Evidence based interventions and novel approaches for oncology practitioners to enhance the QOL and treatment adherence among HNC involve regular screening for distress, educational initiatives, establishment of symptom management clinics, and integration of technology to maintain close communication with patients throughout their treatment journey [44]. Addressing multifaceted QOL challenges faced by HNC patients also necessitates the comprehensive interventions [19]. The CIP implemented to address multitude of issues proved to be effective in improving the QOL, reducing the fatigue, improving the self-efficacy and reducing the psychosocial distress in this distinct group of patient population. Moreover, this benefit was sustained even after six months following treatment. With these promising findings, the CIP also can be implemented in the ongoing routine care of the HNC patients. Hospitals and healthcare facilities may need to allocate resources to implement such tailored interventions to HNC patients. Training healthcare staff to prioritise the needs of patients, strategies such as patient education are crucial [45].

## Strengths and limitations

A comprehensive intervention was required to support and strengthen the resilience of HNC patients as these patients face multitude of problems. To the best of our knowledge this study is the first of its kind attempted to evaluate the effectiveness of comprehensive intervention for the holistic wellbeing of HNC patients. The psychosocial distress scale used in the study was developed based on the findings of focus group discussion among HNC patients emphasizing on the distress experienced during radiotherapy and a systematic review of psychosocial distress of HNC patients during radiotherapy. Hence it was most appropriate for the HNC population. Recruitment and data collection among intervention arm was done only after completing recruitment and collecting two follow-up data from the participants of control arm, and this did not lead into any bias or contamination.

One of the important limitations of the study is that the data were collected amidst of COVID pandemic, which might have impacted the outcome variables, especially psychosocial distress and self-efficacy. Secondly, inclusion of a mix of multiple subsites of HNC such as cancer of oral cavity, pharynx and larynx- which could have potentially unique demands, may pose limitations on generalisation of findings. Thirdly the study was not a randomized control trial and participants were not blinded, hence limits the generalization of findings. Despite best efforts, implementation of the intervention faced few challenges. One of the challenges faced during implementation of the intervention was, difficulty in engaging into the intervention, as the painful procedures (such as tracheostomy and insertion of ryles tube) for few participants were performed on the same day of intervention scheduled. Few other participants experienced fatigue due to chemotherapy schedules which were overlapping with the day of research intervention.

## Conclusion

HNC patients require multimodal treatment modalities and oftentimes, structural, functional, and aesthetic losses result with HNC treatment. From the time of diagnosis, treatment to post treatment follow-up, it is a difficult and lengthy journey which makes HNC one of the most painful cancer experiences mentally and emotionally. The clinical focus during treatment and post-treatment is changing with the emphasis on identifying and minimising treatment side effects and rehabbing the functional deficiencies. The CIP implemented to address multitude of issues proved to be effective in improving the QOL, reducing the fatigue, improving the self-efficacy and reducing the psychosocial distress in this distinct group of patient population.

## Data Availability

As the study participants did not agree to their data to be shared publicly, the data of this study are not publicly available. Participants provided consent only for the result to be written up for publication.
